# New Azaphilones, *Seco*-Chaetomugilins A and D, Produced by a Marine-Fish-Derived *Chaetomium globosum*

**DOI:** 10.3390/md7020249

**Published:** 2009-06-16

**Authors:** Takeshi Yamada, Yasuhide Muroga, Reiko Tanaka

**Affiliations:** Osaka University of Pharmaceutical Sciences, 4-20-1, Nasahara, Takatsuki, Osaka 569-1094, Japan; E-Mail: tanakar@gly.oups.ac.jp (R.T.)

**Keywords:** fungus, marine-fish, Chaetomium globosum, cytotoxicity, chaetomugilins, azaphilones, chemical transformation, alkaline degradation

## Abstract

*Seco*-chaetomugilins A and D were isolated from a strain of *Chaetomium globosum* that was originally isolated from the marine fish *Mugil cephalus*, and their absolute stereostructures were elucidated on the basis of spectroscopic analyses, including 1D and 2D NMR techniques, along with the chemical transformation from known chaetomugilins A and D. *Seco*-chaetomugilin D exhibited growth inhibitory activity against cultured P388, HL-60, L1210, and KB cells.

## 1. Introduction

**Figure 1:**
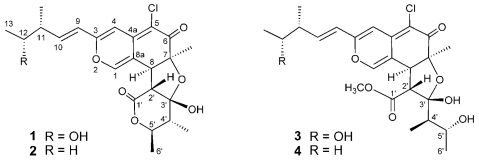
Structures of compounds **1**–**4.**

Marine microorganisms are potentially prolific sources of highly bioactive secondary metabolites that might serve as useful leads in the development of new pharmaceutical agents. Based on the fact that some of the bioactive materials isolated from marine animals have been produced by bacteria, we have focused our attention on new antitumor agents from microorganisms separated from marine organisms [1–5]. As part of this endeavor, we have conducted a search for antitumor compounds from a strain of *Chaetomium globosum* OUPS-T106B-6 that was originally isolated from the marine fish *Mugil cephalus*, and have reported six new cytotoxic metabolites designated as chaetomugilins A (**1**), B, C, D (**2**), E, and F from the culture broth of this fungal strain [6,7]. These compounds are azaphilones and they display various bioactivities, including antimicrobial, nitric oxide inhibitory, gp120-CD4 binding inhibitory, monoamine oxidase inhibitory, and platelet-derived growth factor binding inhibitory properties [7]. An examination of a disease-oriented panel of 39 human cancer cell lines (HCC panel) [8,9] suggested the possibility that the mode of action of chaetomugilins A (**1**), C, and F might be different from that shown by any other anticancer drugs developed to date [7]. Our continuing search for cytotoxic metabolites from this fungal strain yielded two new azaphilones designated as *seco*-chaetomugilins A (**3**) and D (**4**) (Figure 1). *Seco*-chaetomugilin D (**4**) exhibited significant cytotoxic activity against the murine P388 leukemia cell line, the human HL-60 leukemia cell line, the murine L1210 leukemia cell line, and the human KB epidermoid carcinoma cell line. We describe herein the absolute stereostructures and biological activities of these compounds.

## 2. Results and Discussion

*C. globosum* was cultured at 27 °C for six weeks in a medium (100 L) containing 1% soluble starch and 0.1% casein in 50% artificial seawater adjusted to pH 7.4 as reported previously [6,7]. After incubation, the AcOEt extract of the culture filtrate was purified by bioassay-directed fractionation (cytotoxicities against P388 cell line) employing a stepwise combination of Sephadex LH-20, silica gel column chromatography, and reversed-phase HPLC (RP-HPLC) to afford *seco*-chaetomugilins A (**3**) and D (**4**).

**Figure 2:**
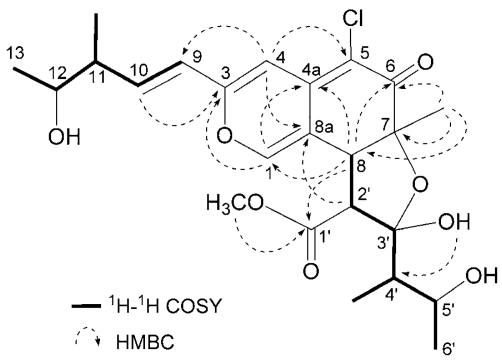
Selected ^1^H-^1^H COSY and HMBC correlations of compound **3**.

*Seco*-chaetomugilin A (**3**) had the molecular formula C_24_H_31_ClO_8_, which was established from the [M+H]^+^ ion in high-resolution fast atom bombardment mass spectrometry (HRFABMS) and the ratio of intensity of isotope ions (MH^+^/[MH+2]^+^). Its IR spectrum exhibited bands at 3,382, 1,736, and 1,642 cm^−1^, which are characteristic of hydroxyl, ester, and conjugated carbonyl groups, respectively. Close inspection of the ^1^H- and ^13^C-NMR spectra (Table 1) of **3** by DEPT and HMQC experiments revealed the presence of four secondary methyls (11-CH_3_, C-13, 4′-CH_3_, and C-6′), one tertiary methyl (7-CH_3_), one ester methyl (1′-OCH_3_), four *sp^2^*-hybridized methines (C-1, C-4, C-9, and C-10) including oxygen-bearing carbon (C-1), six *sp^3^*-methines (C-8, C-11, C-12, C-2′, C-4′ and C-5′) including two oxymethines (C-12 and C-5′), two quaternary oxygen-bearing *sp*^3^-carbon (C-7 and C-3′) including a hemiketal carbon (C-3′), four quaternary *sp^2^*-carbons (C-3, C-4a, C-5 and C-8a) including one oxygen-bearing carbon (C-3), and two carbonyls (C-6 and C-1′). ^1^H-^1^H COSY analysis of **3** revealed two partial structural units as shown by bold-faced lines in Figure 2. The geometrical configuration of the double bond moiety (*Δ*_9, 10_) was deduced to be *trans* from the coupling constants of the olefinic protons (*J*_9,10_ = 15.5 Hz). The connection of these units and the remaining functional groups was determined on the basis of the key HMBC correlations summarized in Figure 2. The connection of a chlorine atom to C-5 was reasonable from its chemical shift (*δ*_C_ 110.0). Thus, the planar structure of **3** was elucidated as shown in Figure 2.

**Scheme 1:**
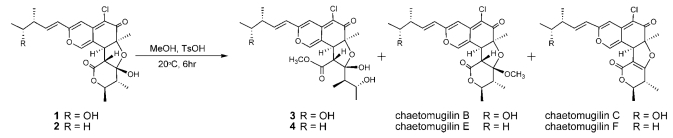
Chemical transformation from **1** and **2** to **3** and **4**.

**Scheme 2:**
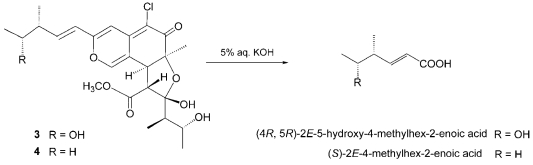
Alkaline degradation of **3** and **4**.

The relative stereochemistry of **3** was examined by conducting NOESY experiments. NOE correlations (8-H/4′-CH_3_ and 7-CH_3_/4′-CH_3_) implied that 8-H is oriented *cis* to the 7-methyl group and C-3′ – C-4′ bond. However, the relative configuration of C-11, C-12, C-2′, C-4′ and C-5′ could not be elucidated. Treatment with *p*-TsOH of chaetomugilin A (**1**) in MeOH gave chaetomugilins B and C as reported previously [6]. This time, the above reaction was carried out on the condition that more amount of *p*-TsOH was used, and then gave **3** together with chaetomugilins B and C (Scheme 1). Product **3** was confirmed to be identical to natural **3** on the basis of IR, UV, and NMR spectra and optical rotations. This result allowed us to assign the absolute configuration of all the asymmetric centers (7*S*, 8*S*, 11*R*, 12*R*, 2′*R*, 3′*R*, 4′*R* and 5′*R*) in **3**. In order to confirm the absolute configuration of C-11 and C-12, alkaline degradation, which is used for the confirmation of flavone character, was applied to compound **3**. The degradation of **3** with 5% potassium hydroxide afforded a carboxylic acid that was identified as (4*R*, 5*R*)-2*E*-5-hydroxy-4-methylhex-2-enoic acid by comparison of spectral data and specific optical rotation with those of carbonic acid obtained from **1** in a similar manner (Scheme 2) [10]. Thus, the absolute configuration at C-11 and C-12 of **3** were supported as *R* and *R*, respectively.

*Seco*-chaetomugilin D (**4**), which contained one oxygen atom less than **3**, was assigned the molecular formula C_24_H_31_ClO_7_ by HRFABMS. The general features of its UV, IR, and NMR spectra (Table 1) closely resembled those of **3** except that the proton signals for H-11 (*δ*_H_2.24, sept), H-12 (*δ*_H_1.42, m), and H-13 (*δ*_H_0.89, t), and the carbon signals for C-10 (*δ*_C_ 146.2), C-11 (*δ*_C_ 38.8), C-12 (*δ*_C_ 29.2), C-13 (*δ*_C_ 11.7), and 11-CH_3_ (*δ*_C_ 19.4) in **4** revealed a chemical shift difference relative to those of **3**. The above evidence implied that the hydroxyl methine at C-12 in **3** was replaced with a methylene in **4**. The planar structure of **4** was confirmed by analyzing ^1^H-^1^H COSY correlations and HMBC correlations. In NOESY experiments, the same NOE correlations (8-H/4′-CH_3_ and 7-CH_3_/4′-CH_3_) as those of **3** were observed. As in compound **3**, treatment with *p*-TsOH of **2** in MeOH gave product **4**, which was confirmed to be identical to natural **4** on the basis of IR, UV, and NMR spectra and optical rotations. The above lines of evidence revealed the absolute stereostructure of **4**. In order to confirm the absolute configuration of C-11, the alkaline degradation was applied to compound **4** by the above method. The degradation of **4** with 5% potassium hydroxide afforded a carboxylic acid that was identified as (*S*)-2*E*-4-methylhex-2-enoic acid by comparison of spectral data and specific optical rotation with those of a commercial sample (Scheme 2) [11]. Thus, the absolute configuration at C-11 was supported as *S*.

During the isolation process, the culture filtrate was extracted with AcOEt, and the time that **1** and **2** were exposed to MeOH on LH-20 and the silica gel column chromatography was very short (6~7 h being the longest time). In addition, chaetomugilins A (**1**) and D (**2**) were stable in MeOH for a few days. As the above reaction, only compounds **3** and **4** were obtained in low yield by treatment with *p*-TsOH of **1** and **2** in MeOH, respectively, but not obtained by the methanolysis using conc. H_2_SO_4_. These evidences confirm that *seco*-chaetomugilins A (**3**) and D (**4**) are not artifacts of **1** and **2**, respectively.

Assays for the growth inhibitory activity of other azaphilones using various cancer cell lines are rarely reported. As a primary screen for antitumor activities, the cancer cell growth inhibitory activities of **3** and **4** were examined using the murine P388 leukemia cell line, the human HL-60 leukemia cell line, the murine L1210 leukemia cell line, and the human KB epidermoid carcinoma cell line. Compound **4** exhibited moderate cytotoxicity to all cell lines (IC_50_: 38.6, 47.2, 53.6, 47.2 μM, respectively). On the other hand, compound **3** showed no growth inhibition against all cell lines. These results implied that the presence of the hydroxyl group at C-12 reduced the activity.

## 3. Experimental

### General

Melting points were determined on a Yanagimoto micro-melting point apparatus and are uncorrected. UV spectra were recorded on a Hitachi U-2000 spectrophotometer and IR spectra, on a JASCO FT/IR-680 Plus. NMR spectra were recorded at 27 °C on Varian UNITY INOVA-500 and MERCURY spectrometers with tetramethylsilane (TMS) as internal reference. FABMS were obtained using a JEOL JMS-700 (Ver. 2) mass spectrometer. Optical rotations were recorded on a JASCO J-820 polarimeter. Liquid chromatography over silica gel (mesh 230–400) was performed at medium pressure. HPLC was run on a Waters ALC-200 instrument equipped with a differential refractometer (R 401) and Shim-pack PREP-ODS (25 cm × 20 mm i. d.). Analytical TLC was performed on precoated Merck aluminum sheets (DC-Alufolien Kieselgel 60 F254, 0.2 mm) with the solvent system CH_2_Cl_2_-MeOH (9: 1), and compounds were viewed under a UV lamp and sprayed with 10% H_2_SO_4_ followed by heating.

### Culture and Isolation of Metabolites

A strain of *C. globosum* was initially isolated from the marine fish *M. cephalus* collected in the Katsuura Bay of Japan in October 2000. The marine fish was wiped with EtOH and its gastrointestinal tract applied to the surface of nutrient agar layered in a Petri dish. Serial transfers of one of the resulting colonies provided a pure strain of *C. globosum*. The fungal strain was cultured at 27 °C for six weeks in a liquid medium (100 L) containing 1% soluble starch and 0.1% casein in 50% artificial seawater adjusted to pH 7.4. The culture was filtered under suction and the mycelia collected were extracted thrice with MeOH. The combined extracts were evaporated *in vacuo* to give a mixture of crude metabolites (42.8 g), the CHCl_3_–MeOH (1:1) soluble fraction of which exhibited cytotoxicity. The culture filtrate was extracted thrice with AcOEt. The combined extracts were evaporated *in vacuo* to afford a mixture of crude metabolites (20.6 g). The AcOEt extract was passed through Sephadex LH-20 using CHCl_3_–MeOH (1:1) as eluent. The second fraction (7.2 g) in which the activity was concentrated was chromatographed on a silica gel column with a CHCl_3_–MeOH gradient as eluent. The CHCl_3_ eluate (1.5 g) was purified by HPLC using MeCN–H_2_O (4:1) as eluent to afford **4** (5.1 mg), and the MeOH–CHCl_3_ (1:99) eluate (1.8 g) was purified by HPLC using MeOH–H_2_O (1:1) as eluent to afford **3** (7.8 mg) together with other chaetomugilins, respectively.

### Seco-chaetomugilin A (**3**)

Pale yellow powder; mp 97–99 *°*C (CHCl_3_–MeOH); [α]_D_^22^ 294.0 (*c* 0.09, EtOH); UV λ_max_ (EtOH)/nm: 292 (log *ɛ* 3.86), 373 (3.94), 405 (4.01); IR *v*_max_ (KBr)/cm^−1^: 3,382 (OH), 1,736 (ester), 1,642 (*α*, *β*-unsaturated carbonyl), 1,563, 1,522 (C=C); FABMS *m*/*z* (rel. int.): 483 ([M+H]^+^, 43.9%), 485 ([M+H+2]^+^, 16.7%); HRFABMS *m*/*z* 483.1782 [M+H]^+^ (calcd for C_24_H_32_^35^ClO_8_: 483.1785); ^1^H- and ^13^C-NMR data are listed in Table 1.

### Seco-chaetomugilin D (**4**)

Pale yellow powder; mp 97–99°C (CHCl_3_–MeOH); [α]_D_^22^ 161.3 (*c* 0.13, EtOH); UV λ_max_ (EtOH)/nm: 291 (log *ɛ* 3.88), 371 (3.80), 411 (3.88); IR *v*_max_ (KBr)/cm^−1^: 3,431 (OH), 1,740 (ester), 1,622 (*α*, *β*-unsaturated carbonyl), 1,564, 1,522 (C=C); FABMS *m*/*z* (rel. int.): 467 ([M+H]^+^, 65.1%), 469 ([M+H+2]^+^, 23.2%); HRFABMS *m*/*z* 467.1840 [M+H]^+^ (calcd for C_24_H_32_^35^ClO_7_: 467.1836); ^1^H- and ^13^C-NMR data are listed in Table 1.

### Derivatization of **3** from **1**

*p*-TsOH (32.7 mg) was added to a MeOH solution (5 mL) of chaetomugilin A (**1**) (23.7 mg) and the reaction mixture was left at room temperature for 6 h. The solvent was evaporated under reduced pressure and the residue was purified by HPLC using MeCN–H_2_O (55:45) as eluent to afford **3** (3.2 mg), chaetomugilin B (6.7 mg), and chaetomugilin C (5.5 mg).

### Degradation of **1** by Potassium Hydroxide

*Seco*-chaetomugilin A (**3**, 15.2 mg) was dissolved in 5% aq. potassium hydroxide (10 mL) and the reaction mixture was stirred for 3 h at 100°C. Then, the reaction mixture was extracted with CHCl_3_ (15 mL). The water layer was adjusted to pH 3.0 with 9% sulfuric acid and re-extracted with CHCl_3_ (10 mL). The organic extract was concentrated to dryness *in vacuo*. The residue was purified by HPLC using MeCN–H_2_O gradient (0:100) – (60:40) as the eluent to afford (4*R*,5*R*)-2*E*-5-hydroxy-4-methylhex-2-enoic acid (0.9 mg). Using the same procedure, chaetomugilin A (26.5 mg), the absolute stereostructure of which was determined already, was treated with 5% aq. potassium hydroxide (20 mL) and purified by HPLC to afford *(4R,5R)-2E-5-hydroxy-4-methylhex-2-enoic acid* (3.1 mg). Colorless oil; [α]_D_^22^ 90.0 (*c* 0.05, EtOH); HRFABMS *m/z*: 145.0867 [M+H]^+^ (calcd for C_7_H_13_O_3_: 145.0865); ^1^H-NMR *δ* ppm (CDCl_3_): 1.12 (3H, d, *J* = 6.5 Hz, 4-CH_3_), 1.19 (3H, d, *J* = 6.2 Hz, H-6), 2.44 (1H, dqd, *J* = 7.5, 6.5, 6.2 Hz, H-4), 3.80 (1H, quint, *J* = 6.2 Hz, H-5), 5.90 (1H, d, *J* = 15.5 Hz, H-2), 7.06 (1H, d, *J* = 15.5, 7.5 Hz, H-3).

### Derivatization of **4** from **2**

Using the same procedure as above with compound **2,** chaetomugilin D (**2**) (22.8 mg) was treated with *p*-TsOH (27.7 mg) in MeOH (8 mL) and the products were purified by HPLC using MeCN–H_2_O (7:3) as eluent to afford **4** (3.5 mg), chaetomugilin E (8.6 mg), and chaetomugilin F (7.2 mg).

### Degradation of **2** by Potassium Hydroxide

*Seco*-chaetomugilin D (**4**, 16.7 mg) was dissolved in 5% aq. potassium hydroxide (15 mL) and the reaction mixture was stirred for 3 h at 100°C. Then, the reaction mixture was extracted with CHCl_3_ (15 mL). The water layer was adjusted to pH 3.0 with 9% sulfuric acid and re-extracted with petroleum ether (15 mL). The organic extract was concentrated to dryness *in vacuo*. The residue was purified by HPLC using MeCN–H_2_O gradient (0:100) – (100:0) as the eluent to afford (*S*)-*2E*-4-methylhex-2-enoic acid (0.7 mg). The physicochemical properties of this carboxylic acid were identical with those of a commercial sample [10].

### Assay for Cytotoxicity to the Cancer Cell Lines

Cytotoxicity of seco-chaetomugilins A (**3**) and D (**4**) were examined by the 3-(4,5-dimethyl-2-thiazolyl)-2,5-diphenyl-2H-tetrazolium bromide (MTT) method. P388, HL-60, L1210, and KB cells were cultured in Eagle’s Minimum Essential Medium (10% fetal calf serum) at 37 °C in 5% CO_2_. The test material was dissolved in dimethyl sulfoxideto give a concentration of 10 mM, and the solution was diluted with the Essential Medium to give concentrations of 200, 20, and 2 μM, respectively. Each solution was combined with each cell suspension (1 × 10^5^ cells/mL) in the medium, respectively. After MTT in phosphate incubating at 37 °C for 72 h in 5% CO_2_, grown cells were labeled with 5 mg/mL buffered saline (PBS), and the absorbance of formazan dissolved in 20% sodium dodecyl sulfate (SDS) in 0.1 N HCl was measured at 540 nm using a microplate reader (Model 450, BIO-RAD). Each absorbance value was expressed as percentage relative to the control cell suspension that was prepared without the test substance with the same procedure as that described above. All assays were performed three times. Semilogarithmic plots were constructed from the averaged data and the effective dose of the substance required to inhibit cell growth by 50% (IC_50_) was determined.

## Figures and Tables

**Table 1:** ^1^H- and ^13^C-NMR data of compounds **3** and **4** in CDCl_3_.

Position		3					4				
		*δ*_H_^a^		*J*/Hz	*δ*_C_		*δ*_H_^a^		*J*/Hz	*δ*_C_	
1		7.58	s		146.2	(d)	7.58	s		145.9	(d)
3					156.6	(s)				157.1	(s)
4		6.49	s		105.1	(d)	6.46	s		104.6	(d)
4a					140.3	(s)				140.5	(s)
5					110.0	(s)				109.7	(s)
6					188.7	(s)				188.6	(s)
7					83.1	(s)				83.1	(s)
8		3.90	d	12.0 (2′)	44.3	(d)	3.90	d	12.0 (2′)	44.3	(d)
8a					114.7	(s)				114.6	(s)
9		6.13	d	15.5 (10)	122.3	(d)	6.03	d	16.2 (10)	120.4	(d)
10		6.58	dd	15.5 (9), 7.8 (11)	141.6	(d)	6.48	dd	16.2 (9), 6.1 (11)	146.2	(d)
11		2.44	dqd	7.8 (10), 6.5 (11-CH_3_), 6.2 (12)	44.2	(d)	2.24	sept	6.1 (10, 12, 11-CH_3_)	38.8	(d)
12		3.80	quint	6.2 (11, 13)	70.9	(d)	1.42	m		29.2	(t)
13		1.19	d	6.2 (12)	20.4	(q)	0.89	t	7.2 (12)	11.7	(q)
7	-CH_3_	1.48	s		25.3	(q)	1.48	s		25.3	(q)
11	-CH_3_	1.12	d	6.5 (11)	14.9	(q)	1.07	d	6.1 (11)	19.4	(q)
1′					169.4	(s)				169.5	(s)
2′		3.24	d	12.0 (8)	53.7	(d)	3.24	d	12.0 (8)	53.7	(d)
3′					107.5	(s)				107.5	(s)
4′		2.01	dq	9.5 (5′), 6.8 (4′-CH_3_)	44.7	(d)	2.01	dq	9.5 (5′), 6.5 (4′-CH_3_)	44.7	(d)
5′		4.33	dq	9.5 (4′), 6.0 (6′)	71.1	(d)	4.33	dq	9.5 (4′), 6.1 (6′)	71.1	(d)
6′		1.30	d	6.0 (5′)	23.4	(q)	1.30	d	6.1 (5′)	23.4	(q)
1′	-OCH_3_	3.74	s		52.1	(q)	3.74	s		52.1	(q)
4′	-CH_3_	0.83	d	6.8 (4′)	13.3	(q)	0.83	d	6.5 (4′)	13.2	(q)
3′	-OH	6.75	br s								
